# Comparing underwater and conventional cold snare polypectomy for colorectal adenomas: Prospective randomized controlled trial

**DOI:** 10.1055/a-2549-0922

**Published:** 2025-04-08

**Authors:** Biao Fu, Xiangrong Zhou, Tian Xiaofeng, Zhi qiang Du, Fei Wang, Da hai Xu, Wang Yue, Wang Jin, Wei-hui Liu

**Affiliations:** 1 Department of Gastroenterology, Jianyang People’s Hospital, Jianyang, Chengdu, China; 2 Department of Internal Medicine I, Jianyang Hospital of Traditional Chinese Medicine, Jianyang, Chengdu, Sichuan Province, China; 3 89669 Department of Gastroenterology, Sichuan Provincial People's Hospital, School of medicine, University of Electronic Science and Technology of China, Chengdu, Sichuan Province, China

**Keywords:** Endoscopy Lower GI Tract, Polyps / adenomas / ..., Tissue diagnosis, Endoscopic resection (polypectomy, ESD, EMRc, ...)

## Abstract

**Background and study aims:**

In this study, we aimed to evaluate efficacy and safety of underwater cold snare polypectomy (UCSP) for treating colorectal adenoma.

**Patients and methods:**

This single-center, prospective, randomized controlled trial screened patients with colorectal adenomas measuring 4 to 9 mm in diameter that were identified through colonoscopies at the Department of Gastroenterology in Jianyang People’s Hospital between April 2022 and October 2023. Patients were randomly assigned to undergo UCSP or cold snare polypectomy (CSP). Both groups underwent narrow-band imaging to determine international colorectal endoscopic morphology of type 2 noncancerous lesions. Following polyp removal, biopsy specimens were collected from the base and margins to assess the completeness of resection.

**Results:**

The study included 227 polyps from 172 patients; median sizes in the UCSP (n = 122) and CSP groups (n=105) were 5 mm and 6 mm, respectively. The R0 (96.7% vs. 86.7%; *P* =0.005) and muscularis mucosa resection rates (68.9% vs. 43.8%; *P* <0.0001) were significantly higher in the UCSP group than in the CSP group. However, operative time for the UCSP group (109.5 s; 86.8–134.3 vs. 110.0 s; 83.5–143.5 *P* =0.890) was not significantly longer than that for the CSP group. Neither group exhibited delayed bleeding or perforations.

**Conclusions:**

UCSP has a high R0 rate for colorectal adenomas measuring 4 to 9 mm.

## Introduction

Colorectal polyps are the main source of colorectal cancer. Endoscopic removal can significantly reduce colorectal cancer incidence 1 . However, 10% to 27% of colorectal cancers are caused by incomplete polyp resection; complete removal of precancerous colorectal lesions thus is crucial for preventing colorectal cancer. Most polyps found during colonoscopy are small (< 10 mm), with extremely low malignancy rates (0.1%) 2 . Removing small colorectal polyps is a primary concern for clinicians and endoscopists. Cold snare polypectomy (CSP) offers better efficacy and safety than with other methods 3 4 5 6 and has become the gold standard for removal of small colorectal polyps (1–9 mm). Electrocautery is not used; therefore, the incidences of postoperative bleeding, post-polypectomy syndrome, and perforation are reduced and operation and resection times are shorter, making it a popular technique in clinical practice. However, hot snare polypectomy (HSP) is associated with a high R0 rate 7 8 9 of 47.3% to 98.2% 5 7 10 . The average submucosa depth was significantly lower in CSP specimens than in HSP and endoscopic mucosal resection (EMR) specimens 11 . Moreover, there is an 11.3% to 36% chance of cold snare defect protrusion (CSDP) after CSP, indicating polyp fragmentation and incomplete mucosal layer resection 12 13 . Therefore, traditional CSP requires further improvement, and more effective and safe removal methods for small colorectal polyps are needed.

Traditional CSP uses air as the intestinal cavity expansion medium. However, because of gravity, the true polyp shape may shrink into a ball, resulting in an unclear polyp boundary display. Consequently, when capturing the polyp, the snare may not achieve a clear view of the cutting-edge margin, which may cause residual tumor tissue on the margin. Polypectomy under underwater endoscopy also has good therapeutic effects 14 . Nevertheless, most studies have focused on larger lesions and require auxiliary measures such as submucosal injection or electrocautery 15 . We hypothesized that underwater CSP (UCSP) could use the magnification and lifting effect of water to clearly display the lesion boundary, remove more of the muscularis mucosa, and improve the R0 rate for small colorectal polyps. Thus, in this study, we aimed to compare UCSP and traditional CSP for small colorectal polyp removal regarding the R0 rate.

## Patients and methods

### Ethics statement

This study was approved by the Institutional Review Board of Jianyang People’s Hospital (approval number 2021 (08)) and registered in the Chinese Clinical Trial Registry (ChiCTR2100044872). All patients provided informed consent.

### Study design

In this prospective study 662 patients with colorectal polyps who underwent colonoscopies at Jianyang People’s Hospital (Sichuan, China) between April 2022 and October 2023 were randomized.

### Inclusion and exclusion criteria

Inclusion criteria were as follows: age 18 to 75 years, single or multiple colorectal polyps measuring 4 to 9 mm in diameter (Paris classification: 0-Ip, 0-Is, 0-Isp, and 0-IIa), and compliance with narrow-band imaging international colorectal endoscopic type 2 classification.

Exclusion criteria were as follows: age < 18 years; presence of familial polyposis or inflammatory bowel disease; history of acute myocardial infarction within the past 6 months; severe heart, liver, kidney dysfunction, or mental disorder; use of aspirin, warfarin, and other anticoagulants or presence of coagulation dysfunction; polyp diameter ≥ 1 cm; participation in other clinical trials within the past 60 days; and pregnancy or sessile serrated lesions.

### Equipment and preoperative preparation

All participants started a liquid diet 1 day before colonoscopy and underwent bowel preparation using 2 L of polyethylene glycol electrolyte powder combined with simethicone. Equipment included a high-definition magnifying endoscope system (CV-290, Olympus, Tokyo, Japan), a conventional endoscope (CF-H290I, PCF-Q260AZI, and PCF-Q260JI, Olympus), biopsy forceps (AMHBFE2.4×1800, Anrei Medical, Zhenjiang, China), and a snare (AMH-SNER241518, Anrei Medical).

### Randomized monitoring

It was impossible to determine whether patients had 4- to 9-mm colorectal polyps before colonoscopy; therefore, a sealed opaque envelope containing a computer-generated random code was opened by the assistant. The doctor was informed of the polypectomy method upon encountering a polyp that met the criteria during the colonoscopy. When multiple eligible polyps were identified in the same patient, the same method was consistently applied for all polypectomies. Lesions that did not meet the inclusion criteria were removed using conventional endoscopic treatment. The pathologist was blinded to the method of endoscopic treatment.

### Endoscopic procedure

**Fig. 1:**
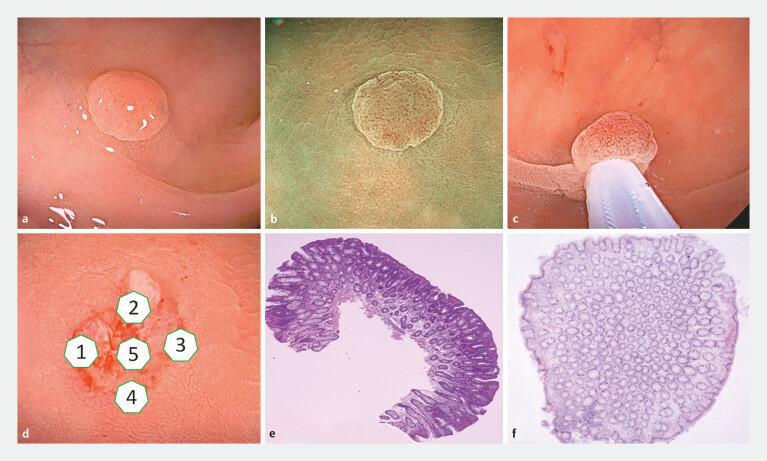
Underwater cold snare polypectomy procedure. **a** . A 5-mm colonic polyp is seen under white light. **b** After the polyp is submerged in water, the surrounding mucosa is smooth, the edges are clear, the light reflection disappears, and the microstructure observed by narrow-band imaging becomes clear. **c** The polyp and the surrounding normal mucosa are captured by a snare. **d** Biopsy specimens are taken from the edges and base of the incision to determine whether any tumor remains. **e** The pathological diagnosis is low-grade dysplasia, which contains more muscularis mucosa. **f** Pathology of the excisional margin biopsy shows no residual tumor cells.

All operators performing endoscopic procedures were experienced professional endoscopists. Each operator had conducted > 5,000 colonoscopies and was proficient in techniques such as CSP, EMR, and endoscopic submucosal dissection. The UCSP procedure was as follows. After finding the target lesion, the digestive tract cavity was filled with sterile water (22 ± 2°C). The polyp was maneuvered to the 6 o’clock position to the extent possible, and a snare was used to capture it (with approximately 1 to 2 mm of normal surrounding mucosa). The snare was gradually reduced until closure was complete. No electrocoagulation was required; instead, the polyp was directly removed by gently pressing the snare against the intestinal wall, which facilitated complete removal (multiple snare resections were not allowed). Subsequently, the endoscope suction button was removed and the suction hole was covered with a finger to prevent tissue damage caused by the suction button. The excised polyp tissue was then suctioned through the endoscope suction channel and collected in a specimen bottle for pathological examination. Following this, biopsies were performed at the base and edge of the polypectomy site in four quadrants—one sample from the base and one from each of the four symmetrical edge points. If a CSDP was observed at the base, it was sampled as a basal biopsy specimen and placed in a separate specimen bottle for examination ( Fig. 1 ). Polyps in the CSP group were removed using conventional CSP, and pathological specimens were obtained following the same protocol as in the UCSP group.

### Basic characteristics of patients and polyps

We evaluated the following indicators: general patient characteristics (age and sex), intestinal score, polyp characteristics (size, location, and Paris classification), and pathological diagnosis results.

Baseline characteristics of patients included in the UCSP and CSP groups were similar in terms of number of polyps detected, sex, age, and Boston Bowel Preparation Scale score ( Table 1 ). The assigned polyps exhibited similar background characteristics.

**Table:** **Table 1** Baseline patient characteristics.

	UCSP	CSP	P value
Patients (n)	89	83	
Sex, n (%)			0.175
Male	54 (60.7)	57 (68.7)	
Female	35 (39.3)	26 (31.3)	
Median age (range, years)	56 (51–62)	57 (51–63)	0.385
BBPS, median (range)	7 (6–7)	7 (6–7)	0.529
BBPS, Boston Bowel Preparation Scale; CSP, cold snare polypectomy; SD, standard deviation; UCSP, underwater cold snare polypectomy.			

Approximately 60% of polyps were classified as type 0-I, whereas 40% were classified as type 0-II. The number of polyps was higher in the left colon than in the right colon. Average size was similar, with polyps measuring 6.2 mm and 5.7 mm in the UCSP and CSP groups, respectively. Over 95% of biopsy types in both groups were low-grade dysplasia. Postoperative pathological examination of biopsy specimens showed that both groups had high-grade dysplasia (HGD; UCSP, n = 2; CSP, n = 5) and HPs (UCSP, n = 3; CSP, n = 2), with no significant differences between the groups ( Table 2 ).

**Table:** **Table 2** Polyp characteristics.

	UCSP	CSP	P value
Morphology, n (%)			0.733
0-Is	37 (30.3)	35 (33.3)	
0-Isp	25 (20.5)	26 (24.8)	
0-Ip	3 (2.5)	2 (1.9)	
0-IIa	57 (46.7)	42 (40)	
Location, n (%)			0.264
Cecum	3 (2.5)	2 (1.9)	
Ascending colon	10 (8.2)	17 (16.2)	
Transverse colon	24 (19.7)	27 (25.7)	
Descending colon	22 (18.0)	15 (14.3)	
Sigmoid colon	44 (36.1)	27 (25.7)	
Rectum	19 (15.6)	17 (16.2)	
Size (mm), n (%)			0.212
4	33 (27.0)	10 (9.5)	
5	38 (31.1)	32 (30.5)	
6	24 (19.7)	39 (37.1)	
7	7 (5.7)	16 (15.2)	
8	14 (11.5)	6 (5.7)	
9	6 (4.9)	2 (1.9)	
Size median (range, mm)	5 (5–7)	6 (5–6)	0.319
Histopathological diagnosis, n (%)			0.172
LGD	118 (96.7)	100 (95.2)	
Tubulovillous adenoma	2 (1.6)	0 (0.0)	
HGD	2 (1.6)	5 (4.8)	
CSP, cold snare polypectomy; HGD, high-grade dysplasia; LGD, low-grade dysplasia; SD, standard deviation; UCSP, underwater cold snare polypectomy.			

### Outcomes

The primary study endpoint was R0 polyp resection, meaning that pathological results of the biopsy specimens at the base and edge of the wound after polypectomy were all negative 9 . When one or more tumor tissues were found in the biopsy specimen at the base or edge of the polyp specimen, it was defined as an incomplete resection (non-R0 resection). Secondary endpoints included resection time (UCSP group: from water injection initiation to specimen retrieval; CSP group: from snare insertion to specimen retrieval), perforation rate (intraoperative and postoperative), bleeding rate (intraoperative [continuous bleeding for ≥ 30 seconds after polypectomy] and postoperative [visible hematochezia within 30 days]), polyp retrieval rate, polyp integrity rate, and CSDP occurrence rate. Resected polyps and biopsy specimens were independently assessed by two pathologists using a blinded method. For disagreements, the pathology was reviewed until a consensus diagnosis was reached. All patients were followed up at our Department of Gastroenterology outpatient clinic 1 week and 1 month after the operation, during which postoperative bleeding and other adverse events (AEs) were recorded.

### Sample size calculation and statistical analysis

The reported range of CSP R0 resection rates for 4- to 9-mm colorectal polyps is 47.3% to 98.2%. Considering clinical reality and expected improvement, we set the CSP R0 resection rate at 72% and hypothesized that the UCSP group could improve to 90%. With an α error level set at 0.05 and controlling the β error level within 0.10, we calculated that at least 98 polyps per group were required. In addition, to account for potential data loss or polyps not meeting the analysis criteria (assuming a 10% exclusion rate), we conservatively increased the sample size for the two groups to a total of 218 polyps to ensure adequacy and robustness of the study.

Numerical data are presented as means ± standard deviations or medians and interquartile ranges. The χ ^2^ test was used to examine relationships between categorical variables, while *t* -tests or Mann-Whitney U tests were employed to assess differences between continuous variables. All statistical analyses were performed using SPSS Statistics for Windows (version 29.0. IBM Corp., Armonk, New York, United States). A significance level of 0.05 was set.

## Results

### Patient recruitment and screening process

**Fig. 2:**
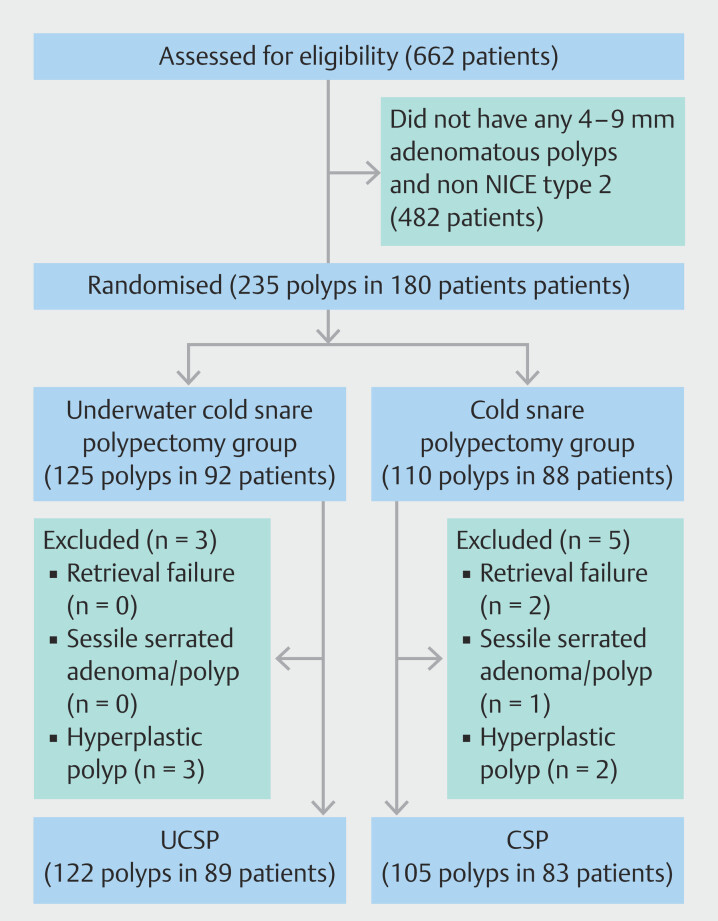
Study flowchart. CSP, cold snare polypectomy; UCSP, underwater cold snare polypectomy.

Between April 2022 and October 2023, 662 patients who underwent colonoscopy were enrolled. Of them, 180 had 235 polyps that met the inclusion criteria. Of these, 92 patients with 125 polyps were assigned to the UCSP group and 88 patients with 110 polyps were assigned to the CSP group. However, three polyps were excluded from the UCSP group because they were hyperplastic. In the CSP group, five polyps were excluded because of retrieval failure (n = 2), sessile serrated adenoma/polyps (n = 1), or hyperplastic polyps (HPs; n = 2). The final analysis included 89 patients with 122 polyps in the UCSP group and 83 patients with 105 polyps in the CSP group ( Fig. 2 ).

### Study endpoints

The R0 resection rate (primary endpoint) was significantly higher in the UCSP group than in the CSP group (96.7% vs. 86.7%, *P* = 0.005), with a higher R0 rate at the base (100% vs. 91.4%, *P* = 0.001); this indicated superior efficacy of UCSP for complete polyp tissue removal. Correspondingly, the resection rate of the muscularis mucosa was significantly higher in the UCSP group than in the CSP group (68.9% vs. 43.8%, *P ≤* 0.0001). There was no significant difference in the submucosal resection rate between the two groups (19.7% vs. 16.2%, *P* = 0.307; Table 3 ). Furthermore, procedure time was comparable between the UCSP and CSP groups (109.5 seconds [86.8–134.3] vs. 110.0 seconds [83.5–143.5], *P* = 0.890). There were no significant between-group differences in terms of AEs, including intraoperative bleeding, postoperative bleeding, and perforation, and no serious AEs occurred. The polyp retrieval rate in the UCSP group was not significantly different from that in the CSP group (100% vs. 98.2%, *P* = 0.218). This indicated that UCSP excels in retrieving resected tissue and preserving the integrity of the polyp specimen, facilitating pathological analysis. There was no significant between-group difference in the proportion of CSDPs (20.5% vs. 14.3%, *P* = 0.147); this indicated that UCSP did not increase risk of polyp fragmentation and residue ( Table 4 ).

**Table:** **Table 3** Primary endpoint.

	UCSP	CSP	Risk difference (95%CI)	P value
R0 resection, n (%)	118 (96.7)	91 (86.7)	4.5 (1.4–14.3)	0.005
Base	122 (100)	96 (91.4)	0.4 (0.38–0.51)	0.001
Containing MM tissue	83 (68.9)	46 (43.8)	2.7 (1.6–4.7)	< 0.0001
Containing SM tissue	24 (19.7)	17 (16.2)	1.3 (0.6–2.5)	0.307
Margins	118 (96.7)	99 (94.3)	1.7 (0.5–6.5)	0.285
CI, confidence interval; CSP, cold snare polypectomy; MM, muscularis mucosa; SM, submucosal; UCSP, underwater cold snare polypectomy.				

**Table:** **Table 4** Secondary endpoints.

	UCSP	CSP	Risk difference (95%CI)	P value
Polyp retrieval rate, n (%)	125 (100)	108 (98.2)	0.5 (0.4–0.5)	0.218
Polyp integrity rate, n (%)			1.6 (0.5–4.8)	0.285
En bloc	116 (95.1)	97 (92.4)		
Multipiece	6 (4.9)	8 (7.6)		
CSDPs, n (%)	25 (20.5)	15 (14.3)	1.5 (0.8–3.1)	0.147
Median resection time (s)	109.5 (86.8–134.3)	110.0 (83.5–143.5)		0.890
Adverse events, n (%)				
Intraoperative bleeding	7 (5.7)	5 (4.8)	1.2 (0.4–4.0)	0.491
Postoperative bleeding	0	0		1.000
Perforation	0	0		1.000
CI, confidence interval; CSP, cold snare polypectomy; CSDPs, cold snare defect protrusions; UCSP, underwater cold snare polypectomy.				

## Discussion

Our findings indicate that UCSP has a higher R0 rate and overall efficacy than conventional CSP for colorectal adenomas, suggesting that UCSP can serve as a viable modality for resecting colorectal adenomas measuring 4 to 9 mm.

The low R0 resection rate in CSP can be primarily attributed to its inability to remove sufficient muscularis mucosa 16 . In specimens removed by CSP, the removal rate for the muscularis mucosa ranges from 35.3% to 57.0% 11 17 , whereas the submucosa inclusion rate is 9% to 29% 11 18 ; both rates are lower than those for HSP and EMR 11 . In this study, the R0 resection rate was lower in the CSP group than in the UCSP group, and the CSP group mainly had residual tumors at the base. This result is similar to that of the study by Maruoka et al 17 , wherein UCSP improved the R0 resection rate by removing more muscularis mucosa. We speculated that because of the lower density of the polyp relative to that of water, polyp immersion in water resulted in a similar “buoyancy” effect on the mucosal layer and submucosa surrounding the lesion. This allowed the snare to remove more mucosa and reduce the residual lesion at the base. Moreover, we observed that after water injection, intestinal peristalsis slowed, and the intestinal mucosa around the polyp was stretched by the water pressure, causing the polyp to be “lifted” by the water. This resulted in a clear display of the polyp boundary and a comprehensive view of the polyp, thereby preventing misjudgments caused by polyp curling and incomplete snaring resulting in residual lesions. In UCSP, the snare can clearly capture an ample amount of normal mucosa surrounding the polyp, thereby minimizing likelihood of residual tissue at the cutting edge. Therefore, compared with CSP using air as the intestinal expansion medium, UCSP can reduce residue at the base and cutting edge, consequently improving the R0 resection rate.

As a new method of endoscopic treatment, UCSP requires more water-injection steps than CSP, which potentially prolongs procedure time. However, our study revealed no significant difference in the procedure time between the UCSP and CSP groups, possibly because water flows through the intestinal cavity, and some polyps were close to one another. With a single water injection, multiple polyps can be consecutively removed. Moreover, once resected underwater, the polyp floats, making retrieval more efficient. The recovery rate for colorectal polyp specimens after CSP was 97.0% to 98.1% 3 19 , and it was challenging to fully recover specimens < 10 mm in diameter. Incomplete and unrecovered specimens cannot provide an accurate pathological diagnosis 20 . Studies have reported that 4% to 60.3% of polyp specimens are fragmented after CSP 12 20 21 22 . Studies have indicated that sampling colorectal polyp specimens directly through the endoscope working channel may lead to tissue damage due to improper handling, thereby affecting accuracy of histological assessment of polyp margin 23 . In our practical experience, we have observed that some polyps tend to become lodged at the suction button during aspiration, increasing risk of incomplete specimens. To address this issue, we retrieved our specimens, we removed the suction button and used the fingertip occlusion method to retrieve the specimens while trying to keep them as intact as possible. Polyp recovery and integrity rates were higher in the UCSP group than in the CSP group. For polyps with unclear edges or unrecovered specimens, doctors suggest repeat colonoscopy. This situation was reduced in the UCSP group, sparing patients from undergoing unnecessary colonoscopies.

The intraoperative bleeding rate for CSP was 0.4% to 9.6% 4 5 10 , and delayed bleeding was significantly reduced after CSP compared with HSP 4 . UCSP did not require high-frequency electrocautery to remove polyps; therefore, it did not cause more complications, such as delayed bleeding and perforation, than HSP 5 . To ensure a negative cutting-edge margin, we removed 1 to 2 mm of normal mucosa surrounding the polyp. However, this method did not increase risk of intraoperative or postoperative bleeding, perforation, or other complications. This was consistent with the results of Abe et al., who found that CSP with an enlarged cutting-edge margin did not increase patient risk 24 . No patient in the study required endoscopic hemostasis, and none experienced perforations. Intraoperative and postoperative bleeding rates were similar between the UCSP and CSP groups, indicating no increase in likelihood of complications. Furthermore, incision bleeding was promptly and clearly identified underwater. In cases in which bleeding persisted, endoscopic metal clip closure of the wound could be performed promptly to effectively stop bleeding and reduce risk of delayed bleeding. Therefore, our results demonstrate that UCSP is safe.

CSDPs often occur during polyp cold snare resections. When using the CSP method to remove colorectal polyps, probability of CSDP occurrence is 11.3% to 36%, and presence of CSDPs indicates polyp fragmentation and incomplete mucosal layer resection 12 13 . Because of the buoyancy effect of water on polyps, UCSP removes a deeper mucosal layer, which may increase the occurrence rate of CSDPs. Our findings showed that incidence of CSDPs was not significantly higher in the UCSP group than in the CSP group. Furthermore, CSDPs did not contain tumor tissue, and their occurrence did not reduce the R0 resection rate. Although the fragmentation rate of the CSDP specimens was higher, the R0 resection rate did not differ between CSDP and non-CSDP specimens 12 . Therefore, UCSP does not increase risk of tumor residues or the CSDP occurrence rate.

This study had some limitations. First, it was a single-center, prospective study. Therefore, it is necessary to increase the sample size, expand the research scope, conduct multicenter randomized controlled trials, and perform long-term clinical follow-up. Second, UCSP requires a large amount of water injection, and the total procedure time is similar to that for CSP. More efficient water-injection equipment could reduce procedure time.

## Conclusions

In conclusion, UCSP had a higher R0 resection rate than conventional CSP for colorectal adenomas measuring 4 to 9 mm. In addition, this endoscopic method did not increase the rate of intraoperative and postoperative bleeding, perforation, or other complications. Therefore, UCSP is a safe and effective technique for resecting small colorectal polyps.
